# L1210 Cells Overexpressing ABCB1 Drug Transporters Are Resistant to Inhibitors of the N- and O-glycosylation of Proteins

**DOI:** 10.3390/molecules22071104

**Published:** 2017-07-03

**Authors:** Lucia Pavlikova, Mario Seres, Milan Hano, Viera Bohacova, Ivana Sevcikova, Tomas Kyca, Albert Breier, Zdena Sulova

**Affiliations:** 1 Institute of Molecular Physiology and Genetics, Centre of Bioscience, Slovak Academy of Sciences, Dúbravska cesta 9, 84005 Bratislava, Slovakia; lucia.pavlikova@savba.sk (L.P.); mario.seres@savba.sk (M.S.); milan.hano@savba.sk (M.H.); viera.bohacova@savba.sk (V.B.); i.sevcikova1@gmail.com (I.S.); tomas.kyca@gmail.com (T.K.); 2 Institute of Biochemistry and Microbiology, Faculty of Chemical and Food Technology, Slovak University of Technology, Radlinského 9, 81237 Bratislava, Slovakia

**Keywords:** L1210 cells, P-glycoprotein, multidrug resistance, N-glycosylation, O-glycosylation, tunicamycin, benzyl 2-acetamido-2-deoxy-α-d-galactopyranoside, ubiquitination

## Abstract

Overexpression of P-glycoprotein (P-gp, drug transporter) in neoplastic cells is the most frequently observed molecular cause of multidrug resistance. Here, we show that the overexpression of P-gp in L1210 cells leads to resistance to tunicamycin and benzyl 2-acetamido-2-deoxy-α-d-galactopyranoside (GalNAc-α-*O*-benzyl). Tunicamycin induces both glycosylation depression and ubiquitination improvement of P-gp. However, the latter is not associated with large increases in molecular mass as evidence for polyubiquitination. Therefore, P-gp continues in maturation to an active membrane efflux pump rather than proteasomal degradation. P-gp-positive L1210 cells contain a higher quantity of ubiquitin associated with cell surface proteins than their P-gp-negative counterparts. Thus, P-gp-positive cells use ubiquitin signaling for correct protein folding to a higher extent than P-gp-negative cells. Elevation of protein ubiquitination after tunicamycin treatment in these cells leads to protein folding rather than protein degradation, resulting at least in the partial lack of cell sensitivity to tunicamycin in L1210 cells after P-gp expression. In contrast to tunicamycin, to understand why P-gp-positive cells are resistant to GalNAc-α-*O*-benzyl, further research is needed.

## 1. Introduction

The development of a specific cell phenotype may include several alterations in the composition of sugars linked to proteins and lipids that form specific glycoms typical for this phenotype. Processes of tissue cell carcinogenesis are frequently associated with the strong remodelation of cell sugar composition [1,2] detectable based on various lectins, which could be used as specific features for differential diagnostics [3,4]. Neoplastic cells could develop resistance to a diverse group of unrelated drugs known as multidrug resistance (MDR), representing a specific cell phenotype secured via several different mechanisms [5] and creating a real obstacle for the effective chemotherapy of cancer [6]. MDR in neoplastic cells has frequently been associated with a massive alteration of cell sugar metabolism/contents [7,8,9]. Drug efflux pumps, members of the ABC transporter gene family, particularly P-glycoprotein (P-gp, also known as ABCB1), are involved in MDR development [10,11]. When expressed in neoplastic cells, P-gp could induce strong resistance (amounting several hundred times) against a large group of structurally unrelated chemicals belonging to a cluster of P-gp substrates [12]. However, several lines of evidence indicated that P-gp may depress the initiation and progression of apoptosis, independently of its drug-efflux activity, through different regulatory pathways [13].

We have described several alterations in the binding of concanavalin A (ConA) to intracellular [14] or plasma membrane [15,16] glycoproteins associated with the overexpression of P-gp in mice L1210 cells. Similar changes in the binding of this lectin onto cell surface areas were observed, independent on the mode of P-gp expression in parental L1210 cells (S) using selection with vincristine as a P-gp inducer (R) [17] or by transfection with a human gene encoding P-gp (T) [15]. Alterations in binding onto the cell surface typical for P-gp-overexpressing R and T cells were also observed for other lectins, such as wheat germ agglutinin (WGA), *Maackia amurensis* agglutinin (MAA) and *Sambucus nigra* agglutinin (SNA) [18]. Changes in ConA, SNA and WGA binding relative to P-gp expression were also observed in human myeloid leukemia SKM-1 (overexpressing P-gp due to selection with vincristine and lenalidomide) and MOLM-13 (overexpressing P-gp due to selection with vincristine) cells [19]. Alterations of cell surface sugars could reflect the presence of large amounts of P-gp in the plasma membrane, which are glycosylated from 145 kDa of unglycosylated polypeptide to 170–180 kDa of final maturated protein [20,21]. The presence of oligosaccharide linked to P-gp was recently attributed to the elevation of α-D-mannosyl and β1-6GlcNAc moieties in P-gp-overexpressing MCF7/ADR cells compared with their P-gp-negative counterpart MCF7 cells [22]. In a previous study, SNA also bound to the oligosaccharide ligands directly presented on P-gp molecules [18]. In contrast, MAA, WGA and *Lycopersicon esculentum* agglutinin (LEA) attached more effectively to the cell surface of P-gp-positive R and T cells than to P-gp-negative S cells [16,18], and ConA, which exerts opposite behavior [15,16], did not recognize the sugar ligands associated with the P-gp molecule. This finding also indicated that the glycosylation of other plasma membrane peptides, distinct from P-gp, is altered when P-gp is overexpressed in L1210 cells. Consistently, we observed lower cellular levels of UDP-glucose in R and T cells than in S cells, indicating a decrease of several cellular transglycosylation reactions, such as glycoprotein formation [14] or glucosylation of ceramides [23]. Tunicamycin (an N-glycosylation inhibitor) has been described as an agent with the potential to reverse P-gp-mediated MDR [24]. Data concerning the effectiveness of O-glycosylation inhibitors, such as benzyl 2-acetamido-2-deoxy-α-d-galactopyranoside (GalNAc-α-*O*-benzyl), are lacking, reflecting the fact that N-glycosylation is a major posttranslational modification of P-glycoprotein [25]. The inhibition of this process by tunicamycin leads to the elevation of ubiquitination and acceleration of proteasomal degradation of P-glycoprotein [26]. However, in previous studies, we described cellular resistance to tunicamycin in P-gp-positive mouse and human leukemia cells [19,27]. Only unglycosylated forms of P-gp (145 kDa) were detected in R and T cells after passaging (1–24 times) in medium containing tunicamycin [27]. However, this form of P-gp was localized in the plasma membrane and exerted P-gp efflux activity. Thus, unglycosylated P-gp, presented as only a P-gp molecular variant in R and T cells after treatment with tunicamycin, may escape from the ubiquitination/proteasomal degradation cascade and become functionally integrated into the plasma membrane. The aims of present paper were: (i) to study cell death effects induced by repeated passaging of S, R and T cells in medium containing tunicamycin or GalNAc-α-*O*-benzyl; (ii) to study the effects of both glycosylation inhibitors on binding of ConA and *Galanthus nivalis* agglutinin (GNA) to cell surface and membrane proteins; (iii) to study the effect of tunicamycin on P-gp ubiqutination in R and T cells.

## 2. Results

### 2.1. Characterization of P-gp Positive Variants of L1210 Cells

Both R and T cells express large amounts of P-gp at the mRNA and protein levels as detected using RT-PCR or western blotting, respectively [15]. The P-gp efflux activity in these cells has previously been demonstrated [15,27] using a calcein/AM retention assay [28]. No measurable amounts of P-gp mRNA and proteins and activity were detected in P-gp-negative S cells [14,15,16,18,19,23,27]. Both R and T cells exert drug resistance to P-gp substrates, such as vincristine, doxorubicin, mitoxantrone and others [19], several hundred times the amount observed in S cells. All these features were periodically controlled for S, R and T cells in our laboratory. Thus, S, R and T cells represent appropriate models for studying specific cellular properties that could accompany the overexpression of P-gp.

### 2.2. Cytotoxic Effect of O- and N-Glycosylation Inhibitors on S, R and T Cells

**Figure 1:**
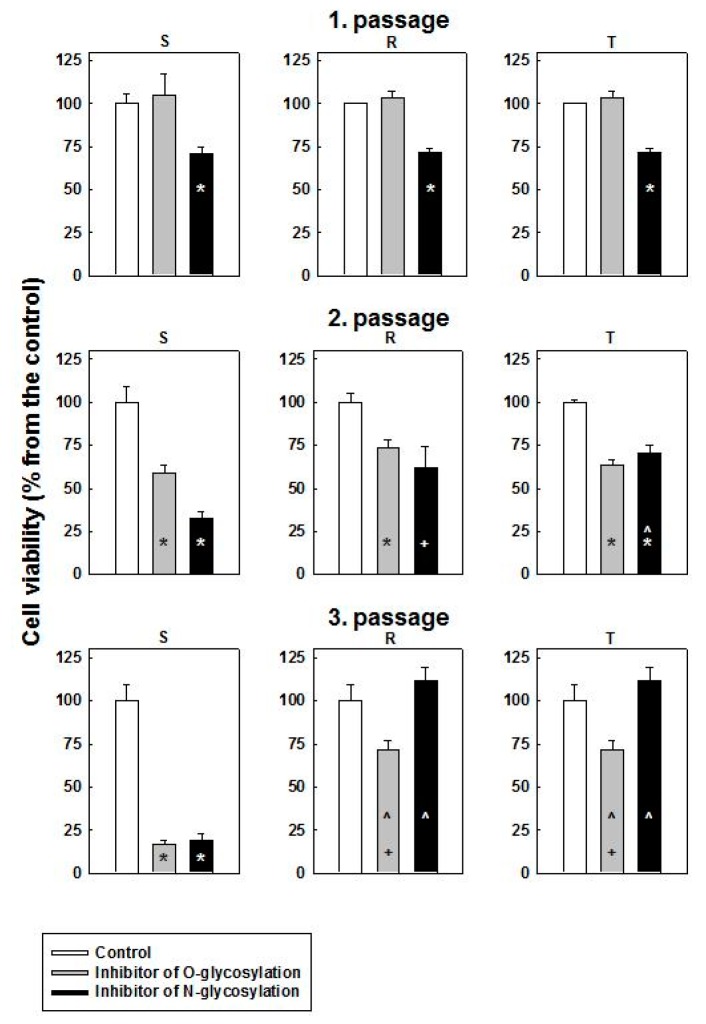
Cell death effects of GalNAc-α-*O*-benzyl (O-glycosylation inhibitor) and tunicamycin (N-glycosylation inhibitor) on S, R and T cells. The cells were passaged 1–3 times in cultivation medium containing either 0.1 mmol·dm^−3^ GalNAc-α-*O*-benzyl or 0.1 μmol·dm^−3^ tunicamycin. Cell viability was assessed using the CASY Model TT Cell Counter (see Materials and Methods). The numbers of viable cells in the control (that were in range 1–2 × 10^6^ cells per cm^3^) were arbitrarily selected as 100%. The data represent the means ± S.E.M. from three independent measurements. Significance: *—values differ from the corresponding control values at *p* < 0.02; +—values differ from the corresponding control values at *p* < 0.05; ^—values differ from the corresponding value for S cells at *p* < 0.02.

To inhibit O- and N- glycosylation, we used GalNAc-α-*O*-benzyl and tunicamycin, respectively. S, R and T cells were passaged 1–3 times in the absence or presence of 0.1 mmol·dm^−3^ GalNAc-α-*O*-benzyl or 0.1 μmol·dm^−3^ tunicamycin in cultivation medium, and subsequently, the viability of the cells was assessed. After the first passage, tunicamycin induced a decrease in the number of viable cells to approximately 70% of the control value in all three variants of L1210 cells (Figure 1).

In contrast to tunicamycin, GalNAc-α-*O*-benzyl did not induce any significant effect on the viability of all three cell variants. The effect of tunicamycin was more pronounced after the second passage, namely, on S cells, in which only one-third of the viable cells were detected compared with the control (Figure 1). R and T cells under these conditions survive considerably better, and more than 60% of viable cells were registered. After the second passage, GalNAc-α-*O*-benzyl induced decreases in cell viability to approximately 40% in all three variants of L1210 cells. Both tunicamycin and GalNAc-α-*O*-benzyl induced a strong decrease of S cell viability after the third passage, and less than 25% of viable cells were detected (Figure 1). In contrast, R and T cells survive much better under these conditions, and the numbers of viable cells exceeded 70% under GalNAc-α-*O*-benzyl treatment or grew similarly under tunicamycin treatment when compared with unaffected control.

After the third passage, R and T cells could be passaged an unlimited number of times in the presence of both inhibitors (data not shown). In contrast, after the third passage, S cells could not be effectively passaged in the presence of either GalNAc-α-*O*-benzyl or tunicamycin. These findings indicated the ability of both P-gp-positive L1210 cells to adapt rapidly in the presence of these inhibitors. Therefore, R and T cells could be considered resistant to GalNAc-α-*O*-benzyl and tunicamycin.

### 2.3. Effect of GalNAc-α-O-benzyl and Tunicamycin on P-gp Glycosylation

**Figure 2:**
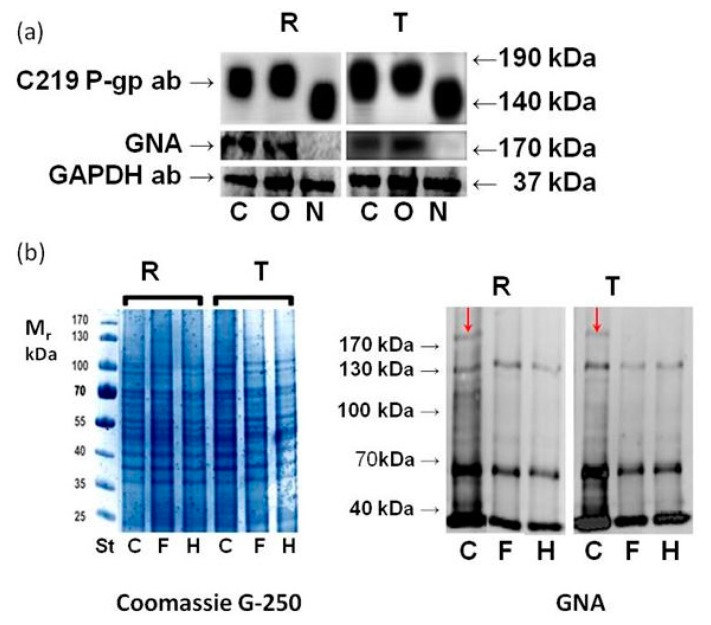
Detection of P-gp glycosylation status in R and T cells. (**a**) Detection of protein bands on Western and Eastern blots using the c219 antibody and GNA in: i. control cells (C) passaged three times in the absence of inhibitors; ii. cells with inhibited O-glycosylation (O) passaged three times in the presence of GalNAc-α-*O*-benzyl (0.1 mmol·dm^−3^); and cells with inhibited N-glycosylation (N) passaged three times in the presence of tunicamycin (0.1 μmol·dm^−3^). The data are representative of three independent measurements. GAPDH was used as a housekeeping protein; (**b**) Detection of cell surface protein deglycosylation in R and T cells using PNGase F (F) and Endo H (H) on Eastern blots using GNA. C represents control cells processed similarly as sub F and H but without enzymes. Red arrows indicate protein bands in the molecular mass region between 170–180 kDa in control cells representing the GNA-detectable P-gp glycoform. The data are representative of three independent measurements. Protein loading was controlled using protein staining with Coomassie G-250 on separate SDS-PAGE gels.

R and T cells contain massive protein bands with molecular masses in the 160–185 kDa region that can be immunodetected using a c219 mouse monoclonal antibody against P-gp (Figure 2a). In contrast, S cells showed only a weak (if any) signal with this antibody (data not shown, but documented elsewhere [15,18,27]).

The application of tunicamycin blocked P-gp glycosylation, and only poorly glycosylated variants of P-gp with considerably decreased relative molecular masses were detected using a c219 antibody compared with unaffected controls (Figure 2a). In contrast, GalNAc-α-*O*-benzyl did not alter the P-gp glycosylation status, and fully glycosylated P-gp variants with molecular masses in a similar range as unaffected control were observed. GNA was described to interact with high mannoses directly associated with P-gp through N-glycosylation [20]. Here, we detected the binding of GNA to protein bands with slightly higher molecular masses than 170 kDa originating from R and T cells either untreated or treated with GalNAc-α-*O*-benzyl. However, R and T cells treated with tunicamycin did not contain protein bands in this molecular mass region that bind to GNA (Figure 2a). To show that GNA binds to this protein band specifically on its N-linked oligosaccharide ligand, we performed deglycosylation using the following enzymes: N-glycosidase F (PNGase F) and Endoglycosidase H (Endo H) (Figure 2b). Quantity of glycoproteins detectable with GNA depresses in R and T cells when either PNGase or Endo H was used for cell treatment visible on Eastern blot. Both enzymes also eliminated the GNA detectability of P-glycoprotein bands, i.e., bands with slightly higher molecular masses than 170 kDa.

### 2.4. Binding of ConA and GNA to Glycoprotein in S, R and T Cells

**Figure 3:**
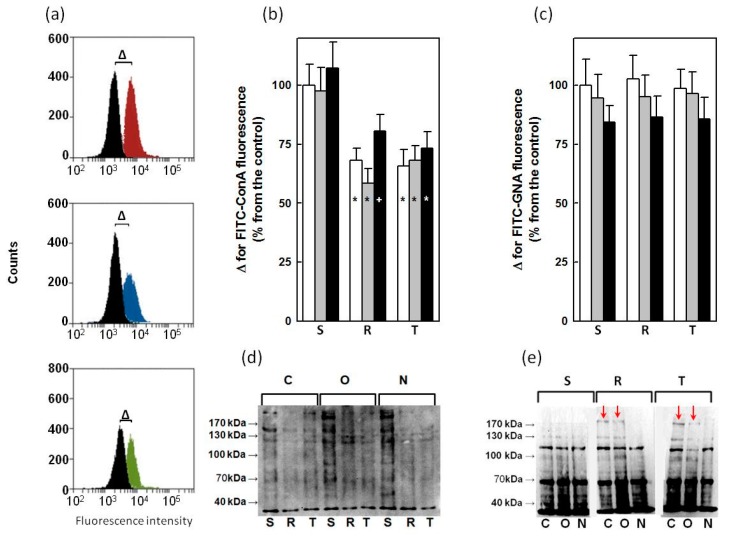
Detection of ConA and GNA binding to glycoproteins in S, R and T cells passaged three times in the absence or presence of either tunicamycin (0.1 μmol·dm^−3^) or GalNAc-α-*O*-benzyl (0.1 mmol·dm^−3^). Panel (**a**): Representative sample of flow cytometry measurement of FITC-ConA binding to S (upper plot), R (middle plot) and T (lower plot) cells. Black histogram – unlabeled S, R and T cells, red histogram – FITC-ConA-labeled S cells, blue histogram FITC-ConA-labeled R cells, and green histogram – FITC-ConA-labeled T cells. Parameter Δ (represents the difference between medians of labeled and unlabeled cells) was ascertained using this measurement. Similar measurements were obtained for S, R and T cells untreated and treated with tunicamycin and GalNAc-α-*O*-benzyl and documented in panels (**b**) (for ConA) and (**c**) (for GNA). Binding of lectin to untreated S cells was arbitrarily selected as 100%. White column—untreated cells; gray—column cell treated with GalNAc-α-*O*-benzyl and black column—cell treated with tunicamycin. Significance: * and +—value significantly differs from the corresponding value for S cells at *p* < 0.02 and *p* < 0.05, respectively. The data represent the means ± S.E.M. of five independent measurements. Panels (**d**) (for ConA) and (**e**) (for GNA) represent Eastern blot identification of glycoproteins in crude membrane fractions isolated from S, R and T cells untreated C, or treated with inhibitor of O-glycosylation (GalNAc-α-*O*-benzyl, O) or N-glycosylation (tunicamycin, N). Data are representative of three independent measurements. Red arrows indicate the P-gp form glycosylated with saccharides that are GNA ligands.

Exposure of saccharide ligands to ConA and GNA on the external surface of S, R and T cells was detected using flow cytometry of cells labeled with lectins labeled with fluorescein isothiocyanate (FITC-ConA, FITC-GNA). Untreated and cells treated with tunicamycin or GalNAc-α-*O*-benzyl were used for these measurements. A typical sample of this measurement is documented on Figure 3a. Either unlabeled or cells labeled with the respective lectins were counted, and differences (Δ) between medians of fluorescence intensities obtained for labeled and unlabeled cells were considered as a measure of cell surface binding to FITC-ConA or FITC-GNA.

The parental P-gp-negative variant of L1210 cells (S) bound to ConA more effectively as their P-gp-positive counterparts R and T cells (Figure 3b). More pronounced binding of ConA to glycoprotein in the crude membrane fraction isolated from S cells (compared with R and T cells) was also detected in Eastern blots (Figure 3d). In contrast to ConA, GNA labels the surfaces (Figure 3c) and glycoproteins in crude membrane fractions isolated from S, R and T cells to a similar extent. Neither tunicamycin nor GalNAc-α-*O*-benzyl was altering the binding of ConA (Figure 3b) or GNA (Figure 3c) onto the surfaces of S, R and T cells, significantly. Similarly, the treatment of S, R and T cells with tunicamycin or GalNAc-α-*O*-benzyl did not induce any remarkable changes of ConA and GNA binding to glycoproteins in crude membrane fractions compared with untreated control, except for tunicamycin-induced GNA binding to oligosaccharides directly associated with P-gp. The detection of the P-gp glycosylated form with affinity to GNA is shown in Figure 3e. This specific glycosylation could be decreased after treatment with tunicamycin but not GalNAc-α-*O*-benzyl. Any other such pronounced changes in glycosylation status under treatment with these two inhibitors were not observed on Eastern blots when either ConA or GNA were used for detection.

### 2.5. Effect of Tunicamycin on Protein Ubiquitination in R and T Cells

**Figure 4:**
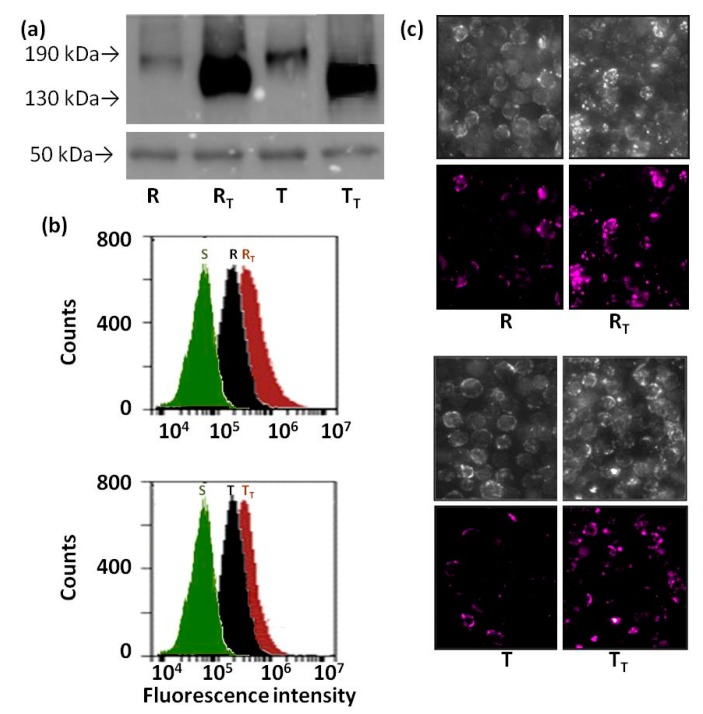
Ubiquitination of proteins in R and T cells passaged four times in the presence or absence of tunicamycin. Panel (**a**) detection of P-gp in immunoprecipitates obtained with rabbit polyclonal anti-ubiquitin antibody (IgG). Whole cell lysates were immunoprecipitated, and subsequently P-gp was detected using Western-blotting with a c219 antibody. The detection of rabbit IgG heavy chain (50 kDa) using an anti-rabbit antibody was included as an internal standard; Panel (**b**)—detection of ubiquitinated material on the cell surface of R and T cells using a rabbit anti-ubiquitin polyclonal antibody and goat anti-rabbit antibody conjugated to Alexa Fluor 660 as primary and secondary antibodies, respectively; Panel (**c**) represents the same experiments as panel (**b**) but evaluated in InCell confocal image detection. Upper panels represent cell visualization in light microscopy mode, and the lower panels represent detection of Alexa Fluor 660 fluorescence. S represents sensitive cells cultivated four times in the absence of tunicamycin. R, T and R_T_, T_T_ represent either R or T cells passaged four times in the absence or presence of tunicamycin (0.1 μmol·dm^−3^), respectively. Data in each panel are representative for three independent experiments.

Total protein lysates isolated from R and T cells cultivated for four passages in the presence or absence of tunicamycin were immunoprecipitated using an anti-ubiquitin polyclonal antibody. The immunoprecipitate was further analyzed using western blotting with a c219 antimouse antibody (Figure 4a). Small amounts of c219-detectable proteins with molecular weights below 190 kDa were detected in immunoprecipitates originated from control R and T cells passaged four times in the absence of tunicamycin. Immunoprecipitates obtained from R and T passaged four times in the presence of tunicamycin contained significant amounts of c219-detectable proteins, but with smaller molecular weights than observed for tunicamycin-unaffected cells (Figure 4a). Both R and T cells contain larger amounts of ubiquitinated materials on the cell surface compared with S cells (Figure 4b). The ubiquitination of R and T cell surfaces was slightly elevated after four passages in the presence of tunicamycin. Differences in cell surface ubiquitinylation after four passages in the presence of tunicamycin are also shown in Figure 4c for both R and T cells.

## 3. Discussion

Both P-gp-positive variants of L1210 cells (R and T) are resistant to inhibitors of O- and N-glycosylation (GalNAc-α-*O*-benzyl and tunicamycin). Resistance of P-gp-positive cells to GalNAc-α-*O*-benzyl during repeated passages of cells in the presence of this inhibitor (Figure 1) represents a rather unexpected result. We do not have a precise explanation for such resistance yet, but the following ideas could help resolve this question in future studies. This inhibitor was described to blocked O-glycosylation of mucins [29]. In cancer cells, mucins are overexpressed on the entire cell surface [30]. Decreases in mucin synthesis by GalNAc-α-*O*-benzyl improve pancreatic cancer cell sensitivity to 5-fluorouracil [30]. The expression of mucin 4 in melanoma cells leads to resistance to 2-deoxyglucose and several P-gp substrates, such as taxol, doxorubicin, and vinblastine [31]. However, the downregulation of P-gp was observed in mucin 4-positive melanoma cells compared with mucin-negative counterparts accompanied with a higher retention of rhodamine 123 within cells expressing mucin 4 compared with mucin-negative cells [31]. Thus, the resistance of mucin 4-positive cells to P-gp substrate is secured by a mechanism distinct to P-gp and its drug efflux activity. Taken together, the overexpression of P-gp in neoplastic cells may be associated with alterations in O-glycosylated cell surface proteins, including mucins, and this alteration may be responsible for the reduced cell sensitivity to the O-glycosylation inhibitor GalNAc-α-*O*-benzyl.

The resistance of P-gp-positive leukemia cells to tunicamycin has recently been described for mouse L1210 and human SKM-1 or MOLM-13 cells [19,27]. The mechanism of this resistance is not fully understood and could be explained in the following ways:

(a) Tunicamycin may be a substrate for P-gp efflux and therefore could effectively be eliminated from the intracellular volume of P-gp-positive cells. While this idea has previously been discussed [32], direct evidence for such P-gp activity is lacking. However, the fact that tunicamycin also blocks the glycosylation of several proteins, including P-gp, in P-gp-positive cells (as shown in Figure 2 and described elsewhere [27,33,34]) is contradictory to this speculation. Moreover, resistance of R and T cells to tunicamycin could not be reversed by verapamil (a known P-gp inhibitor) even if this substance completely blocked P-gp efflux activity [27].
(b) Tunicamycin induced the global inhibition of glycoprotein synthesis (Hiss et al., 2007) associated with the elevation of immature proteins cell content leading to endoplasmic reticulum stress and apoptosis [35]. P-gp-positive cells with altered regulation of apoptosis initiation and progression [13,17] could survive better under this condition.
(c) The application of tunicamycin increases the cellular level of UDP-N-acetylglucosamine, associated with different features of cell damage visible using electron microscopy, namely, in the membranes of the endoplasmic reticulum and nuclei [34]. Significantly decreased intracellular levels of UDP-sugars and UDP-glucose have been detected in P-gp-positive R and T cells compared with P-gp-negative S cells [14,23]. Therefore, the resistance of P-gp-positive L1210 cells to tunicamycin may reflect the decreased levels of UDP-sugars in these cells.

Tunicamycin also abolish specific P-gp N-glycosylation detectable with GNA either in R or T cells (Figure 2 and Figure 3). GNA ligand specificity required high mannose trees containing mannoses linked with α(1–2), α(1–3) or α(1–6) and a nonreducing terminal D-mannose residue [20,36]. This N-glycosylation of P-gp in R and T cells could be enzymatically eliminated by both PNGase F and Endo H (Figure 2). PNGase F cleaves all asparagine-linked complex, hybrid, or high mannose oligosaccharides unless the core GlcNAc contains an α-1,3-fucose [37]. At least two N-acetylglucosamine sequentially attached to asparagine residues are necessary for PNGase F catalysis [38]. Endo H cleaves the bond in the glycosylation core of the N-linked oligosaccharide between two N-acetylglucosamines directly proximal to the asparagine residue on high-mannose and hybrid, but not complexed, glycans [39]. This cleavage generates a truncated sugar molecule with one N-acetylglucosamine residue remaining on the asparagine residues of the glycosylation site. Taken together, at least a portion of the P-gp glycoforms contains N-linked oligosaccharides with structural consensus for GNA binding and both glycosidase reactions. Previously, we described the detection of glycosylated P-gp via SNA [18]. This finding indicated the presence of one branched oligosaccharide-capped hybrid with terminal sialic acids on the P-gp molecule [9]. However, large variability of oligosaccharides linked to P-gp molecules could be expected because there are at least 150 known isoforms of P-gp, which could present a different glycome [20]. Nevertheless, oligosaccharides linked to mouse P-gp expressed in R cells and human P-gp expressed in T cells are exerted from the point of recognition by GNA, PNGase F and Endo H in a similar manner.

Differences in cell binding capacity for ConA between S cells that bound larger amounts and R or T cells that bound lower amounts are shown in Figure 3. This finding is consistent with previous findings [15,16]. In contrast, any such visible alterations could not be detected for GNA binding to S, R and T variants of L1210 cells. Neither tunicamycin nor GalNAc-α-*O*-benzyl induced detectable changes in ConA or GNA binding to the surfaces of S, R and T cells measured using flow cytometry (Figure 3). Similar behavior, with the exception of tunicamycin, induced the blocking of GNA binding to P-gp as detected on Eastern blots (Figure 3).

Several lines of evidence indicate that the lack of P-gp N-glycosylation induced by tunicamycin leads to the elevation of ubiquitination and subsequent proteasomal degradation of unglycosylated proteins [26]. This effect may result in the depression of P-gp-mediated drug resistance [24]. However, we detected an unglycosylated form as the only form of P-gp in R and T cells treated with tunicamycin, and moreover, this form was localized to the plasma membrane and exerted drug efflux activity measured using a calcein/AM retention assay [27]. This finding indicated altered ubiquitination and subsequent processes in both P-gp-positive variant of L1210 cell. Ubiquitin, a polypeptide of 76 amino acids in length (M_r_ 8500 kDa) can be covalently attached to lysines of target proteins either as a monomer or as a lysine-linked polymer. The ubiquitinated proteins showed an increase in molecular weight compared to the non-modified form, and the precise molecular weight of the modified proteins depends on the number of ubiquitins on the modified proteins [40]. On a simplistic level, monoubiquitination has largely been linked to chromatin regulation, protein sorting, and trafficking, whereas polyubiquitination has been associated with protein signaling and clearance through proteasomal or autophagic degradation [41]. Ubiquitin contains seven lysine residues (K6, K11, K27, K29, K33, K48 a K63) and any of these residues could be used for the linking of another ubiquitin molecules as part of polyubiquitin chain formation [42]. Polyubiquitination through different lysines has been proposed to alter ubiquitin signaling. For example, polyubiquitination on K48 forms a conventional chain, leading to degradation in proteasomes. In contrast, polyubiquitination through K63 is also abundant, representing a signal for kinase activation, DNA- repair processes and vesicle trafficking [43]. Taken together, the destiny of ubiquitinated proteins depends on the number of bound ubiquitins in the chain and, in the case of polyubiquitination, on special structural features of the polyubiquitin chain. P-gp in R and T cells were ubiquitinated as detected on the western blot obtained by immunoprecipitation with an anti-ubiquitin antibody (Figure 4a). This ubiquitination is rather low because the final protein band did not exceed a molecular mass of 190 kDa, i.e., the improvement of molecular mass due to linkage of ubiquitin is not large. When anti-ubiquitin immunoprecipitates were processed for R and T cells treated with tunicamycin, much larger amounts of ubiquitinated P-gp were observed by western blotting. However, the molecular mass of this protein band was lower than that obtained for untreated cells. Thus, there was no such elevation of P-gp molecular mass to detect P-gp polyubiquitination in R and T cells under tunicamycin treatment. R and T cells contain larger amounts of ubiquitin immunoreactive material on the surface than S cells (Figure 4b). The latter fact indicated that ubiquitin signaling in the proper folding of plasma membrane proteins is better developed in P-gp-positive than in P-gp-negative cells. Treatment with tunicamycin induced the further elevation of ubiquitin on the cell surface that could be detected using flow cytometry or confocal microscopy. Taken together, R and T cells use ubiquitin signaling for correct protein folding, and the elevation of protein ubiquitination after tunicamycin treatment in these cells leads to protein folding rather than protein degradation. Finally, this finding may represent crucial points that cause at least the partial resistance of R and T to tunicamycin.

## 4. Materials and Methods

### 4.1. Cell Culture Conditions

The following three L1210 cell variants were used in this study: (i) S-drug-sensitive parental cells obtained from Leibniz-Institut DSMZ-Deutsche Sammlung von Mikroorganismen und Zellkulturen GmbH (Braunschweig, Germany) ACC-123; (ii) R—P-gp-positive drug-resistant cells that overexpress P-gp after selection with vincristine [44] obtained from Gedeon Richter Co., (Budapest, Hungary); and (iii) T—P-gp-positive drug-resistant cells that overexpress P-gp following stable transfection with the P-gp gene [15], using the Addgene plasmid 10957 (pHaMDRwt), a retrovirus encoding the full-length P-gp cDNA [45]. The cells (S, R and T; inoculums 1 × 10^6^ cells) were cultured in 4 cm^3^ RPMI 1640 media with l-glutamine (1 mg cm^−3^), 4% fetal bovine serum and 1 μg cm^−3^ gentamycin (all purchased from Gibco, Langley, OK, USA) in a humidified atmosphere with 5% CO_2_ and air at 37 °C for 48 h in the absence or presence of either tunicamycin (0.1 μmol·dm^−3^) or GalNAc-α-*O*-benzyl (0.1 mmol·dm^−3^). This procedure was termed as passage and was repeated three times. Numbers of viable cells after each passage were counted using a CASY Model TT Cell Counter (Roche Applied Sciences, Madison, WI, USA). R cells were cultured for two passages without VCR prior to the experiments.

### 4.2. Western and Eastern Blot Procedures

P-gp and other membrane glycoproteins were detected by either western blot using specific antibody or Eastern blot using lectins (ConA and GNA) in crude membrane fractions isolated from S, R and T cells three times passaged in the absence or presence of either tunicamycin (0.1 μmol·dm^−3^) or GalNAc-α-*O*-benzyl (0.1 mmol·dm^−3^). Total cell proteins were fractionated to crude membrane fraction (CMF) and cytosolic fractions (CF) using the ProteomeExtract Subcellular Proteome Extraction Kit (Calbiochem, San Diego, CA, USA) according manufacturer’s protocol. Proteins from CMF (30 μg per line) were separated via sodium dodecyl sulfate polyacrylamide electrophoresis (SDS-PAGE) on polyacrylamide gradient gels (8–16%) using protocol published by Laemmli [46]. Proteins were subsequently transferred by electroblotting onto nitrocellulose membranes (GE Healthcare Europe GmbH, Vienna, Austria) using protocol published by Towbin et al. [47]. The C219 anti-P-gp monoclonal antibody (Calbiochem) in dilution 1:75 was used to detect P-gp by western blotting. An anti-mouse secondary antibody conjugated to horseradish peroxidase was used as secondary antibody (GE Healthcare Europe GmbH) in dilution 1:2000. For glycoprotein detection by Eastern blots, ConA and GNA conjugated with biotin (EY Laboratories Inc., San Mateo, CA, USA), and avidin conjugated with horseradish peroxidase (Sigma-Aldrich, San Diego, CA, USA) was used. Visualization of bands was processed with the aid of the ECL detection system (GE Healthcare Europe GmbH) and a CF 440 scanning system (Kodak, Rochester, NY, USA) . To provide an internal control GAPDH was detected in CF (30 μg protein per line) by rabbit polyclonal antibodies against GAPDH (Santa Cruz Biotechnology, Dallas, TX, USA) in dilution 1:200 and goat anti-rabbit IgG conjugated with horseradish peroxidase (Santa Cruz Biotechnology), in dilution 1:500 served as primary and secondary antibody, respectively. Coomassie blue (Sigma-Aldrich) staining of separate polyacrylamide gels was also used to verify the accuracy of protein loading for Eastern and western Blots.

### 4.3. Deglycosydation of Membrane Proteins with PNGase and Endo H

Membrane proteins 2 mg/cm^3^ in crude membrane fractions isolated from S, R and T cells (see previous chapter) were thermally denatured in 0.1 mol dm^−3^ phosphate buffer (pH 7.2) containing 20 mmol·dm^−3^ EDTA, 50 μmol·dm^−3^ SDS and 2.6 mm^3^ per dm^3^ β-mercaptoethanol for 15 min at 100 °C. Subsequently, the samples were cooled and 66 μmol·dm^−3^ of NP-40 (Sigma-Aldrich) were added. Either PNGase or Endo H (0.5 U, Roche Applied Sciences) were added to the sample and incubated for 14 h at 37 °C. The samples were analyzed via Eastern blotting with GNA (see previous section).

### 4.4. Detection of ConA and GNA Binding to the Surface of S, R and T Cells by Flow Cytometry

After three passages in the absence or presence of either tunicamycin (0.1 μmol·dm^−3^) or GalNAc-α-*O*-benzyl (0.1 mmol·dm^−3^), the cells were harvested by centrifugation and washed three times with phosphate-buffered saline (PBS containing 137 mmol·dm^−3^ NaCl, 2.7 mmol·dm^−3^, 10 mmol·dm^−3^ Na_2_HPO_4_ and 1,8 mmol·dm^−3^ KH_2_PO_4_), resuspended in RPMI medium without fetal bovine serum (5 × 10^5^ cells/cm^3^) and incubated for 60 min with FITC-labeled ConA or GNA (EY Laboratories Inc.) at a concentration of 1 mg/L in a humidified atmosphere supplemented with 5% CO_2_ at 37 °C [18]. After incubation, the cells were washed three times with PBS, and specifically labeled cells were counted using a BD Accuri C6 flow cytometer (BD Bioscience, San Jose, CA, USA).

### 4.5. Immunoprecipitation of Proteins by Antiubiquitin Antibody

R and T cells were passaged four times in the presence or absence of tunicamycin (0.1 μmol·dm^−3^). Subsequently, the cells were harvested, and whole-cell lysates were prepared by homogenization in SoluLyse (Thermo Scientific, Darmstadt, Germany) according to the manufacturer’s instructions. The protein concentration was determined using the Lowry assay and used for immunoprecipitation. The proteins (80–100 μg) from R and T cells were adjusted to a final volume of 300 μL using 50 mmol·dm^−3^ Tris-HCl (pH 7.0) and anti-ubiquitin antibody (rabbit polyclonal sc-9133, Santa Cruz Biotechnology, final dilution 1:500) was added. After 2 h of incubation at 4 °C, 20 μL of protein A/G PLUS-agarose (Santa Cruz Biotechnology) was added, and the mixture was incubated overnight at 4 °C. Subsequently, the precipitates were pelleted by centrifugation (10 min; 10,000 rpm; 4 °C) and washed two times with 50 mmol/L Tris-HCl buffer. The precipitates were applied on 8% SDS-PAGE and subsequently the proteins were transferred by electroblotting onto nitrocellulose membrane, and the presence of P-gp in the immunoprecipitate was detected using the same antibodies as described for the Western blot procedure. Signal of rabbit IgG heavy chain originated from anti-ubiquitin antibody was detected as internal standard using goat anti-rabbit antibody conjugated with horseradish peroxidase (Santa Cruz Biotechnology).

### 4.6. Detection of Ubiquitin on Cell Surface of S, R and T Cells by Confocal Microscopy and Flow Cytometry

R and T cells were passaged four times in the presence of 0.1 μmol/dm^3^ tunicamycin. S cells were passaged in a parallel experiment but in the absence of tunicamycin. Subsequently, the cells were harvested, washed three times with PBS, resuspended in RPMI medium without fetal bovine serum (5 × 10^5^ cells in dm^3^) but with 5% of defatted BSA (Sigma Aldrich) and incubated for 240 min with anti-ubiquitin antibody (described in previous section) in a humidified atmosphere supplemented with 5% CO_2_ at 37 °C. After incubation, the cells were washed three times with RPMI medium containing 5% BSA and subsequently left to interact with secondary antibody (Goat anti-Rabbit IgG linked with Alexa Fluor 660, A21074, Thermo Fisher Scientific, Bratislava, Slovak Republic). The labeled cells were either counted in the BD Accuri C6 flow cytometer or were evaluated using the InCell 2000 Analyzer (GE Healthcare Europe GmbH).

## 5. Conclusions

P-gp-positive variants of L1210 cells are also resistant to tunicamycin and GalNAc-α-*O*-benzyl. This resistance could be recognized as a side effect of P-gp overexpression in these cells and may be independent of P-gp efflux activity. Tunicamycin inhibits P-gp specific N-glycosylation. In addition to this specific feature, both GalNAc-α-*O*-benzyl and tunicamycin did not induce detectable changes in the content of glycoprotein ConA and GNA ligands in either crude membrane fraction proteins or on the cell surface. However, ConA interacts with S cell glycoproteins in the crude membrane fraction or on the cell surface at a higher extent than in R and T cells. R and T cells contain higher amounts of ubiquitin linked with cell surface protein than S cells. Tunicamycin induced elevation of P-gp ubiquitination, but with no pronounced increase of molecular mass, excluding the likelihood of massive polyubiquitin labeling. Therefore, in these cells, P-gp is not sufficiently ubiquitinated for recognition in proteasomal degradation and continues maturation to a fully active membrane efflux pump. While the GalNAc-α-*O*-benzyl resistance of R and T cells is not understood, the resistance of these cell variants to tunicamycin could reflect alterations in ubiquitination mechanisms. The fact that the surface of R and T cells in the absence of tunicamycin contained higher amounts of ubiquitin indicated by the utilization of protein ubiquitination during cell maturation at a higher extent than in S cells. The acceleration of protein ubiquitination in these cells after tunicamycin treatment leads to protein folding rather than protein degradation.
